# Impact of Antiretroviral Therapy on the Incidence of Tuberculosis: The Brazilian Experience, 1995–2001

**DOI:** 10.1371/journal.pone.0000826

**Published:** 2007-09-05

**Authors:** Abraham Miranda, Meade Morgan, Leda Jamal, Kayla Laserson, Draurio Barreira, Guida Silva, Joseney Santos, Charles Wells, Patricia Paine, Denise Garrett

**Affiliations:** 1 The Global AIDS Program, United States Centers for Disease Control and Prevention, Atlanta, Georgia, United States of America; 2 Division of TB Elimination, United States Centers for Disease Control and Prevention, Atlanta, Georgia, United States of America; 3 Centro de Referência e Treinamento DST/AIDS, STD/AIDS Program of the State of Sao Paulo, State of Sao Paulo Department of Health, Sao Paulo, Brazil; 4 Office of the Director, Office of the Global Health Coordinator, United States Centers for Disease Control and Prevention, Atlanta, Georgia, United States of America; 5 Programa Nacional DST/AIDS, Brazil Ministry of Health, Brasília, Brazil; 6 Programa Nacional de Controle da Tuberculose, Brazil Ministry of Health, Brasília, Brazil; 7 International Union Against TB and Lung Diseases, Paris, France; University of Ioannina School of Medicine, Greece

## Abstract

**Background:**

The human immunodeficiency virus (HIV) fuels tuberculosis (TB) epidemics. In controlled clinical trials, antiretroviral therapy (ART) reduces TB incidence in HIV-infected patients. In this study we determine if, under programmatic conditions, Brazil's policy of universal ART access has impacted TB incidence among HIV-infected patients.

**Methods:**

We abstracted clinical information from records of HIV-infected patients managed in the public sector in 11 Brazilian states between 1/1/1995 and 12/31/2001. Case ascertainment (TB and HIV) utilized guidelines (with added stringency) published by Brazil's Ministry of Health. We determined TB incidence and hazards ratio (HR) for ART-naïve and ART-treated [including highly active ART (HAART)] patients employing Cox proportional hazards analysis.

**Results:**

Information from 463 HIV-infected patients met study criteria. The median age of the study population was 34 years, 70% were male, and mean follow-up to primary endpoints—TB, death, and last clinic visit—was 330, 1059, and 1125 days, respectively. Of the 463 patients, 76 (16%) remained ART-naïve. Of the patients who never received HAART (n = 157) 81 were treated with ART non-HAART. Of the patients who received any ART (n = 387), 306 were treated with HAART (includes those patients who later switched from ART non-HAART to HAART). Tuberculosis developed in 39/463 (8%) patients. Compared to HAART- and ART non-HAART-treated patient groups, TB incidence was 10- (p<0.001) and 2.5-fold (p = 0.03) higher in ART-naïve patients, respectively. The median baseline absolute CD4+ T-lymphocyte count for patients who developed TB was not significantly different from that of patients who remained TB free. In multivariate analysis, the incidence of TB was statistically significantly lower in HAART-treated [HR 0.2; 95% (CI 0.1, 0.6); p<0.01] compared to ART naïve patients. A baseline CD4+ T-lymphocyte count <200 cells/mm^3^ [HR 2.5; (95% CI 1.2, 5.4); p<0.01], prior hospitalization [HR 4.2; (95% CI 2.0, 8.8); p<0.001], prior incarceration [HR 4.1; 95% CI 1.6, 10.3); p<0.01], and a positive tuberculin skin test [HR 3.1; (95% CI 1.1, 9.0); p = 0.04] were independently and positively associated with incident TB.

**Conclusion:**

In this population-based study we demonstrate an 80% reduction in incident TB, under programmatic conditions, in HAART-treated HIV-infected patients compared to ART-naïve patients.

## Introduction

Tuberculosis (TB) is the leading cause of morbidity and mortality in people living with the human immunodeficiency virus (HIV) [1]–[1]. Patients infected with HIV are at increased risk, up to 10% per year, of reactivating latent *Mycobacterium tuberculosis* infection, and of accelerated progression to TB disease soon after infection [5]–[8]. In addition, HIV appears to increase the rate of TB re-infection and recurrent disease [9], [10]. During the last decade, TB case notification rates have risen sharply in areas of high HIV prevalence, such as sub-Saharan Africa, largely attributable to the HIV epidemic [8], [11]–[13]. The World Health Organization (WHO) recommends that affected countries implement collaborative programmatic TB/HIV activities, including antiretroviral therapy (ART), as part of the health sector response to this syndemic [14], [15].

Before 1996, ART consisted of an antiretroviral (ARV) agent (primarily a nucleoside reverse transcriptase inhibitor, or NRTI) given alone or in combination with another ARV agent. Thereafter, the introduction of highly active ART (HAART), consisting of three ARV agents [including NRTI, non-nucleoside transcriptase inhibitors (NNRTI) and protease inhibitors (PI)], has had a significant impact on reducing the incidence of opportunistic infections (OI) in HIV-infected patients [16]–[19]. In prospective clinical studies, HAART has been shown to reduce the incidence of TB by as much as 80% [20]–[23]. Further, epidemiological models of TB in a theoretical setting of ART accessibility have demonstrated considerable impact of HAART on TB incidence, but these estimates have relied on assumptions such as ART being initiated early in the course of HIV infection and very high levels of treatment adherence being attained [24]. The true impact of ART on TB among people living with the human immunodeficiency virus under normal program conditions, however, has not been previously reported.

In 1996, Brazilian federal law established a mandate granting universal access to ART for all eligible HIV-infected individuals [25]. In a nation where the high national TB burden [estimated (2005) all case TB incidence and prevalence of 60/100,00 population (pop)/yr and 76/100,000 pop, respectively] points to significant community transmission, where the adult (15–49 yrs) HIV prevalence (2005) among TB patients is 14%, and where the vast majority of TB and HIV patients are managed in the public sector, Brazil's public policy guaranteeing universal ART access has important implications for TB control [25]. According to the WHO, in 2005, 9529 new TB cases and 2281 TB deaths (all forms) were attributable to HIV, despite the fact that 86% of known HIV-infected adults eligible for ART in Brazil were receiving this therapy [26], [27]. However, up to 64% of prevalent HIV infections remain undiagnosed in the country, according to estimates (2002) by Brazil's Ministry of Health (MoH), *Programa Nacional de DST e AIDS* (national program for STI and AIDS—unpublished data). This estimate suggests that, in addition to the need for improved HIV counseling and testing access, the total burden of HIV infection in Brazil represents a large reservoir of people at increased risk of TB [20]–[23].

This population-based study sought to evaluate the impact of universal access to ART on TB disease incidence in HIV-infected patients managed in the public health sector in Brazil.

## Methods

### Study population

The study population comprised a retrospective cohort of HIV-infected individuals receiving care in public HIV treatment facilities in Brazil.

The study sample size, 474, was calculated to discern a minimum protective effect of 35% among HAART-treated compared to ART-naïve patients with a 95% confidence level and 80% statistical power [28]. In 2000, 11% of all new TB cases in adults, globally, occurred in persons infected with HIV; and we used this figure to estimate the frequency of TB among ART-naïve HIV-infected individuals in our sample size calculation [13]. The study population was drawn from systematically selected public HIV treatment facilities in Brazil using population-proportional-to-size sampling, cluster size of 20 patients, and selection of records at facilities by generating random number lists.

### Inclusion Criteria

The medical records of patients were abstracted if (a) the initial facility visit was recorded to be between January 1, 1995 and December 31, 2001, and (b) HIV was diagnosed and confirmed during that period. Only an HIV Western Blot, indirect immunofluorescence testing, or evidence of viremia (by polymerase chain reaction) were accepted as confirming the diagnosis. Medical records of patients were excluded if there was evidence that the patient had attended clinic only once during the study period, were <18 years of age, pregnant, or wards of the state.

### Data abstraction

Data abstractors, trained by investigators, utilized standardized and validated data collection tools to transcribe information from patient records. Information collected included patient demographics; historical information about TB, incarceration, hospitalization, injection drug use (IDU), and homelessness; presumptive HIV transmission route; date of and vital status at last recorded facility visit; HIV-related diseases included in the WHO clinical staging system [29]; baseline and follow-up CD4+ T-lymphocyte counts and plasma viral load; tuberculin skin testing (TST) and acid-fast bacilli (AFB) smear and culture results; diagnostic x-ray and pathology reports; and, if administered, date and duration of prescribed ART, TB treatment, co-trimoxazole (CTM) prophylactic treatment, and isoniazid preventive therapy (IPT).

### Antiretroviral therapy

According to the Brazil national HIV clinical guidelines, in brief, all patients, regardless of symptomatology, are eligible for ART if their CD4+ T-lymphocyte count (in Brazil determined by flow cytometry) is <200 cells/mm^3^; or if patients develop HIV-associated clinical manifestations such as *Pneumocystis jiroveci* (*carinii*) pneumonia [25], [30]. Antiretroviral therapy is considered in asymptomatic patients with CD4+ T-lymphocyte counts between 200 and 350 cells/mm^3^ according to the clinical and laboratory evolution of their immunological features (e.g., presence of OI and measured plasma viral load) and patient characteristics (e.g., adherence and motivation) [30].

The ARV agents and ART regimens received by patients between the initial facility visit and a study primary endpoint (see below) are listed in Tables 1 and 2. We classified ART as, (1) HAART: a combination of (a) 2 NRTI+1 PI, or (b) 2 NRTI+1 NNRTI, or (c) 1 NRTI+1 NNRTI+1 PI; (2) ART non-HAART: any single ARV agent or combination not defined as HAART; and (3) ART-naïve: no ART received.

**Table 1:** Cumulative list of antiretroviral agents available in public HIV treatment facilities in Brazil between January 1, 1995 and December 31, 2001.

NRTI*	NNRTI**	Protease inhibitors
Abacavir	Delavirdine	Amprenavir
Didanosine	Efavirenz	Atazanavir
Lamivudine	Nevirapine	Indinavir
Stavudine		Lopinavir/ritonavir
Zalcitabine		Nelfinavir
Zidovudine		Ritonavir
Zidovudine/Lamivudine		Saquinavir

* Nucleoside reverse transcriptase inhibitor

** Non-nucleoside reverse transcriptase inhibitor

**Table 2:** List of the most commonly prescribed classes of and specific antiretroviral agents and their combinations used in HAART and ART non-HAART ART regimens among patients attending public HIV treatment facilities in Brazil (n = 463) between January 1, 1995 and December 31, 2001.

Most commonly prescribed ART regimens	No. of times used, at least once, during the study period
**HAART**	
**Drug classes** *: NRTI**×2+PI***×1	221
NRTI×2+NNRTI**×1	119
**Specific** ^	
zidovudine+lamivudine+indinavir	81
zidovudine+lamivudine+nelfinavir	49
zidovudine+lamivudine+nevirapine	46
stavudine+lamivudine+indinavir	29
zidovudine+didanosine+indinavir	25
zidovudine+lamivudine+efavirenz	24
**ART non-HAART**	
**Drug classes**: NRTI×2	208
NRTI×1	61
**Specific**:	
zidovudine+didanosine	133
zidovudine	51
zidovudine+lamivudine	51
zidovudine+zalcitabine	48
stavudine+lamivudine	17
didanosine+stavudine	11

* A total of 36 unspecified ART regimens (HAART = 22 and ART non-HAART = 14) were cumulatively used for at least one day, 780 times during the study period^∼^

** See footnotes, Table 1

*** Protease inhibitors (includes ritonavir-boosted regimens)

^A total of 177 specific ART regimens were cumulatively used for at least one day, 1421 times during the study period^∼^

∼ Note that the number of ART regimens cumulatively used during the study period exceeds the total study population because, for those on ART, more than one regimen may have been prescribed during the study period

### Tuberculosis case definition

We classified a case of TB based on a modified (i.e., more stringent) case definition as published in the Brazil national TB guidelines as, (a) confirmed: *Mycobacterium tuberculosis* isolated in culture from a clinical specimen, (b) probable: AFB identified by *Kinyoun* or *Ziehl-Neelsen* staining in a clinical specimen, and (c) presumed: documented evidence of an abnormal chest x-ray consistent with TB disease, caseous granulomatous reaction in a tissue specimen, or the prescription of anti-TB treatment by a physician [31]. Pulmonary TB was defined as TB disease involving the lungs, irrespective of whether disease was present in other locations; whereas extrapulmonary TB was defined as TB disease excluding the lungs.

### CD4+ T-lymphocyte count

A baseline CD4+ T-lymphocyte count was defined as the result of this test at initial facility visit; whereas, the pre-diagnostic CD4+ T-lymphocyte count was defined as the result of the test closest to the time of TB diagnosis.

### Data analysis

Data analysis was performed using Epi Info v.6 and v.3.1 (CDC, Atlanta, GA, USA) and SAS v.8.01 (SAS Institute, Cary, NC, USA). Observations were censored at the date of TB diagnosis, date of last clinic visit, date of patient death, or January 1, 2002 (i.e., primary endpoints). We excluded from analysis patients in whom TB was diagnosed within 30 days of the recorded initial clinic visit because the immunorestorative effect of ART [we used the immune reconstitution syndrome (IRIS) as a marker for this] would not be expected to develop in most HIV-infected TB patients within the first 4 weeks (range 0 to12 weeks) of initiating ART [32], [33].

Cox proportional hazards modeling was used to calculate hazards ratios, 95% confidence limits, and p-values in univariate screening and multivariate analysis. Variables associated with a p-value≤0.2 in univariate screening were considered for the multivariate model. In the univariate screening, patients whose treatment was switched from ART non-HAART to HAART during the study period were considered in the HAART group irrespective of the amount of time they would eventually spend on HAART. In multivariate analysis, the proportional amount of time on ART non-HAART and HAART were treated as time-dependent co-variables. All two-way interactions were tested. Manual backward stepwise regression was used to construct the final model. A p-value≤0.05 was considered statistically significant.

### Ethical considerations

The protocol for this study was approved by Brazil's MoH ethical review committee and determined as non-research by the National Center for HIV, STD, and TB Prevention of the US Centers for Disease Control and Prevention. Patient medical records kept at facilities contained routinely-collected clinical information and only this information was obtained. The study included no patient contact, and the abstraction forms and databases created for this study recorded no patient identifiers. Data abstractors were trained in issues of confidentiality and privacy by investigators.

## Results

Of 1002 records reviewed in 29 facilities in 11 Brazilian states, 307 (31%) were not included because 203/307 (66%), 63/307 (21%), and 41/307 (13%) individuals were not HIV-infected, lacked an HIV confirmatory test, and their initial facility visit occurred outside of the date parameters for inclusion, respectively. Of the remaining 695 (69%) records, 174 (25%) met exclusion criteria [73/174 (42%), 57/174 (33%), 12/174 (7%), and 32/174 (18%) represented individuals who attended clinic only once, were pregnant, were <18 years of age, or met more than one exclusion criteria, respectively]. An additional 58/695 (8%) records were excluded due to a recorded TB diagnosis occurring within 30 days of the recorded initial facility visit. Thus, 463/695 (66%) records comprised the study population, the median age of which was 34 years (range 18–71), and 70% were male (Table 3).

**Table 3:** Socio-demographic characteristics, by TB status, of patients attending public HIV treatment facilities in Brazil (n = 463) between January 1, 1995 and December 31, 2001.

Characteristic		Number with feature/total* (%)	No. with TB (%)	p-value
**Sex**	Male	322/459 (70)	30 (9)	
	Female	137 (30)	9 (6)	NS
**Age groups (years)**	18–29	128/452 (28)	13 (10)	
Median age 34 years	30–34	113 (25)	8 (7)	NS
(range 18–72 years)	>34	211 (47)	18 (9)	
**State where patient treated**	Bahia (BA)	16/463 (3)	1 (6)	
	Goias (GO)	36 (8)	2 (6)	
	Minas Gerais (MG)	16 (3)	2 (6)	
	Mato Grosso do Sul (MS)	17 (4)	0 (0)	
	Pernambuco (PE)	13 (3)	0 (0)	
	Parana (PR)	12 (3)	1 (8)	NS
	Rio de Janeiro (RJ)	62 (13)	6 (10)	
	Rio Grande do Norte (RN)	15 (3)	1 (7)	
	Rio Grande do Sul (RS)	17 (4)	1 (6)	
	Santa Catarina (SC)	13 (3)	3 (23)	
	São Paulo (SP)	246 (53)	22 (9)	
**Mode transmission**	Ever injection drug			
	Use (IDU)	61/461 (13)	10 (16)	0.02
	Heterosexual**	107/232 (46)	10 (9)	NS
	Male sex with male**	136 (59)	12 (9)	NS

* Denominators vary due to missing data in the patient medical record for each characteristic (missing values deleted from analysis)

** Each subcategory (i.e., heterosexual and MSM) includes the subset of male patients who were reported as bisexual (n = 45 of 152 heterosexual males), and therefore, the sum of the numerators of both subcategories is greater than the denominator

Except for patients with a history of injection drug use (IDU; *Χ^2^*, p = 0.02), no statistically significant differences were observed between the patients who eventually developed TB and those who did not develop TB with respect to sex, age, mode of sexual transmission, or state where the patient was clinically managed (Table 3). For whom the information was available in the patient record (i.e., denominators vary), 229 and 116 of 379 patients presented with a baseline viral load of ≥10,000 and ≥100,000 copies/ml, respectively; and 263/429 (61%) presented with a baseline CD4+ T-lymphocyte <200 cells/mm^3^. Two-hundred and twenty-three of 271 (82%) patients presented or developed WHO stages 3 or 4 clinical disease. Recorded modes of HIV transmission (not mutually exclusive) included 136/232 men who had sex with men (MSM), 61/461 with history of IDU, 232/297 with multiple sexual partners (heterosexual and MSM), 151/244 with HIV-positive partner contact, and 12/331 with prior blood transfusion.

**Figure 1:**
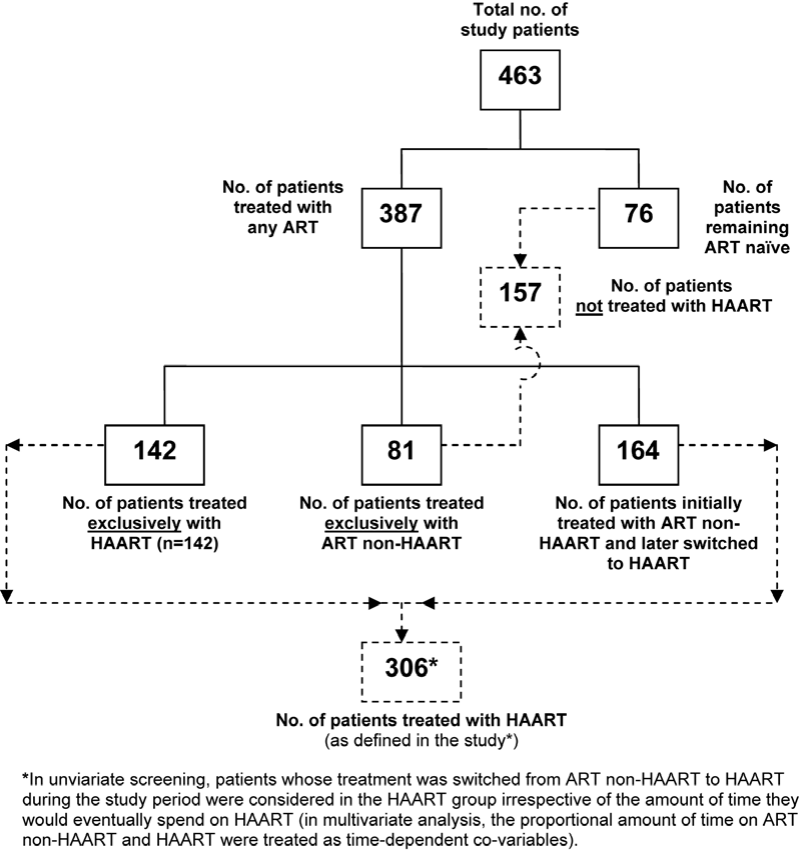
Breakdown of study patients attending public HIV treatment facilities in Brazil (n = 463) between January 1, 1995 and December 31, 2001 by intervention (HAART, ART non-HAART), and non-intervention (ART naïve).

Except for the 76/463 (17%) patients who remained ART-naïve, all patients who initiated any ART (n = 387) did so at some point after their initial recorded facility visit. At the time of the primary endpoints, 245 [patients treated exclusively with ART non-HAART (n = 81)+those initially treated with ART non-HAART and later switched to HAART (n = 164)] and 306 [patients treated exclusively with HAART (n = 142)+those initially treated with ART non-HAART and later switched to HAART (n = 164)] patients had contributed survival time to ART non-HAART and HAART regimens, respectively (Fig. 1). One hundred-fifty seven patients received no HAART. The drug classes and specific agents that composed the most commonly prescribed ART regimens in this study are listed in Table 2. At least one dose of CTM was prescribed for 204/441 (46%) patients.

Nine (23%) confirmed, 8 (21%) probable, and 22 (56%) presumed TB diagnoses (n = 39) were made during the study period. Twenty-three (59%) patients had pulmonary TB, the remaining 16 (41%) had extrapulmonary disease. Eighteen (46%) TB cases occurred in ART-naïve patients. The median baseline absolute CD4+ T-lymphocyte count for patients who developed TB was not significantly different from patients who remained TB free (111 cells/mm^3^ vs. 253 cells/mm^3^, respectively. *Χ* ^2^, p>0.05). However, a higher proportion of patients with TB had median baseline CD4+ T-lymphocyte counts <200 cells/mm^3^ compared to patients without TB (57% vs. 37%, respectively. *Χ* ^2^, p = 0.01). The median pre-diagnostic (TB) CD4+ T-lymphocyte count was 134 cells/mm^3^ (range 6–821 cells/mm^3^). The median time elapsed between the pre-diagnostic CD4+ T-lymphocyte count and the diagnosis of TB was 73 days (range 0–572).

Mean duration of follow-up from the initial recorded facility visit to a primary endpoint was 330, 1059, and 1125 days for TB diagnosis , patient death, and last clinic visit, respectively. Of the 43 (9% of study population) patient deaths recorded during the study period, 3 had been diagnosed with TB disease (*Χ* ^2^: p>0.05).

We analyzed potential risk factors for TB disease in the study population (Table 4). Patients with a baseline CD4+ T-lymphocyte count of <200 cells/mm^3^ had more than twice the incidence of TB disease than those with a baseline CD4+ T-lymphocyte count >200 cells/mm^3 ^{hazards ratio (HR) 2.3; [95% confidence interval (CI)] 1.2, 4.4}. A history of IDU, incarceration, hospitalization, and prior TB were also associated with a statistically significantly higher incidence of TB disease (Table 4). No statistically significant difference was observed in the incidence of TB disease among patients with baseline (i.e., at initial visit to the facility) WHO clinical stages 3 and 4 vs. 1 and 2, [HR 1.4; (95% CI 0.5, 3.6)].

**Table 4:** Univariate Cox proportional hazards screening of risk of TB among patients attending public HIV treatment facilities in Brazil (n = 463) between January 1, 1995 and December 31, 2001.

Characteristic* ^,^ ^		No. with feature/Total^^ (%)	No. with TB#	TB Incidence per 100 person-years	Hazard ratio (95% CI)	p-value
**Antiretroviral therapy**	Any ART	387/463 (84)	21	1.9	0.2 (0.1, 0.3)	<0.01
	**ART-naïve**	76 (16)	18	13.4		
	ART non-HAART exclusively	81/157 (52)	10	4.9	0.4 (0.2, 0.9)	0.03
	**ART-naïve**	76 (48)	18	13.4		
	HAART	306/387 (80)	11	1.2	0.1 (0.1, 0.2)	<0.001
	**ART-naïve**	76 (20)	18	13.4		
	HAART	306/387 (79)	11	1.2	0.3 (0.1, 0.6)	<0.01
	**ART non-HAART exclusively**	81 (21)	10	4.9		
**Baseline WHO clinical stage**	3 or 4	223/271 (82)	29	5.0	1.4 (0.5, 3.6)	NS
	**1 or 2**	48 (12)	5	3.4		
**Baseline CD4+ T-lymphocyte count (cells/mm^3^)**	≥200	166/429 (39)	21	2.0	2.3 (1.2, 4.4)	0.01
	**<200**	263 (61)	16	5.1		
	≥350	283/429 (66)	8	1.8	2.0 (0.9, 4.4)	NS
	**<350**	146 (34)	29	3.6		
**Baseline HIV serum viral load (copies/ml)**	≥10,000	229/379 (60)	12	2.7	1.0 (0.5, 2.2)	NS
	**<10,000**	150 (40)	18	2.7		
	≥100,000	116/379 (31)	12	3.9	1.7 (0.8, 3.5)	NS
	**<100,000**	263 (69)	18	2.3		
**Co-trimoxazole prophylactic treatment**	Yes	204/441 (46)	13	2.5	0.6 (0.3, 1.2)	NS
	**No**	237 (54)	26	3.9		
**Tuberculin skin test positive (TST+)**	Positive	21/122 (19)	4	7.0	3.0 (0.9, 10.7)	NS
	**Negative**	101 (81)	6	2.1		
**Isoniazid preventive Treatment (in TST+)**	Yes	11/21 (52)	0	5.5	0.0 (0.0, -)	0.06
	**No**	10 (48)	3	12.0		
**Prior history of TB**	Yes	30/397(8)	25	11.9	4.0 (1.7, 9.2)	<0.01
	**No**	367 (92)	7	3.0		
**Prior contact with TB patient**	Yes	15/113 (13)	2	6.0	7.1 (1.0, 50.3)	NS
	**No**	98 (87)	2	0.7		
**History of ever having been hospitalized**	Yes	163/409 (40)	27	7.0	5.1 (2.4, 10.9)	<0.001
	**No**	246 (60)	9	1.3		
**History of ever having been incarcerated**	Yes	18/245 (7)	6	16.8	5.0 (2.0,12.4)	<0.001
	**No**	227 (93)	26	3.3		
**Ever homeless**	Yes	10/279 (4)	2	13.7	4.5 (1.0, 19.3)	NS
	**No**	269 (96)	19	2.7		
**Ever IDU**	Yes	61/461 (13)	10	6.4	2.5 (1.2, 5.1)	0.01
	**No**	400 (87)	29	2.6		

* Denominators vary due to missing data from the patient medical record for characteristic (missing values deleted from analysis)

^Reference value appears bolded on the second line of each characteristic. Note that those patients who received ART non-HAART and were later switched to HAART were classified as patients having received HAART (see data analysis section of Methods)

^^The word ”Total” in the “No. with feature/Total” column refers to the denominator for each particular characteristic (e.g., 81 out of a total of 157 patients who did not receive HAART were treated with ART non-HAART exclusively)

# The total number of patients who developed TB (n = 39) varies for each characteristic according to whether or not patients were excluded from analysis due to incomplete information (in the patients' medical record) for that characteristic

In univariate screening (Table 4), ART-naïve patients had a statistically significantly higher incidence of TB disease than those receiving any ART [HR 5.0; (95% CI 3.3, 10.0); p<0.01]. Statistically significant protective effects against incident TB disease was observed for both ART non-HAART (exclusively) [HR 0.4; (95% CI 0.2, 0.9), p = 0.03] and HAART [HR 0.1; (95% CI, 0.1, 0.2), p<0.001].

In multivariate analysis, controlling for type of ART intervention, a baseline CD4+ T-lymphocyte count <200 cells/mm^3^, IPT, and prior history of TB, hospitalization, incarceration and IDU (Table 5), only HAART (vs. ART naïve) was associated with a statistically significant reduction in TB disease incidence [HR 0.2 (95% CI 0.1, 0. 6); p<0.01]. The reduction in TB disease among patients receiving ART non-HAART (vs. ART naïve) did not reach statistical significance [HR 0.4; (95% CI 0.2, 1.1); p = 0.08]. A baseline CD4+ T-lymphocyte count <200 cells/mm^3^ [HR 2.5; (95% CI 1.2, 5.4); p<0.01], prior hospitalization [HR 4.2; (95% CI 2.0, 8.8); p<0.001] , prior incarceration [HR 4.1; 95% CI 1.6, 10.3); p<0.01], and a positive tuberculin skin test [HR 3.1; (95% 1.1, 9.0); p = 0.04] were independently and positively associated with incident TB. All two-way interaction terms were tested and none were found to be statistically significant (i.e., p>0.1).

**Table 5:** Adjusted* hazards ratio of the risk of tuberculosis among patients attending public HIV treatment facilities in Brazil (n = 463) between January 1, 1995 and December 31, 2001 in a Cox proportional hazards model.

Characteristic	Adjusted hazards ratio for TB disease (95% CI)	p-value
HAART (*vs*. ART-naïve)	0.2 (0.1, 0. 6)	<0.01
ART non-HAART exclusively (*vs*. ART-naïve)	0.4 (0.2, 1.1)	0.08
Baseline CD4+ T-lymphocyte count <200 cells/mm^3^	2.5 (1.2, 5.4)	<0.01
Tuberculin skin test positive	3.1 (1.1, 9.0)	0.04
History of ever having been hospitalized	4.2 (2.0, 8.8)	<0.001
History of ever having been incarcerated	4.1 (1.6, 10.3)	<0.01

* All two-way interactions were tested

Note: neither the term co-trimoxazole prophylactic treatment nor IPT were statistically significant in the final model

**Figure 2:**
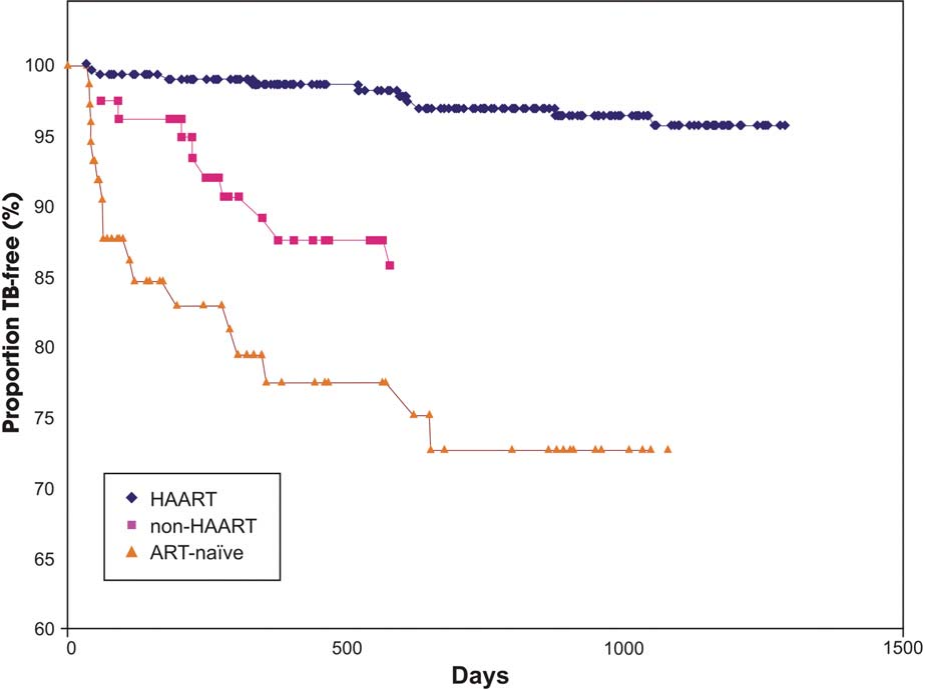
Kaplan-Meier survival curve* of the proportion of patients attending public HIV treatment facilities in Brazil (n = 463) between January 1, 1995 and December 31, 2001 who remained TB free at primary study endpoint (i.e., TB, death, or last clinic visit) over the time course (in days) of the study period, by intervention (HAART, ART non-HAART), and non-intervention (ART naïve).

Ninety-five percent, 86%, and 69% of patients who received HAART, ART non-HAART , and remaining ART naïve, respectively, remained free of TB disease at primary endpoints (p<0.001 for all three situations, when evaluated together; see Figure 2).

The mortality rate of the study population at study end was 0.24 per 100-person years of follow-up. The proportional mortality of severely immunocompromised TB patients (CD4+ T-lymphocyte<200 cells/mm^3^) at 6 and 12 months after starting TB treatment, was 0.8% and 1.1%, respectively.

## Discussion

In this population-based retrospective cohort analysis of adult HIV-infected patients managed under normal programmatic conditions in public HIV treatment facilities in Brazil, we observed a statistically significantly lower (80%, p<0.01) incidence of TB in patients prescribed HAART compared to patients who remained ART-naïve. This effect of HAART remained statistically significant even when controlling for the patients' baseline immunological status, and other important characteristics and interventions (Tables 4 and 5). Though treatment with ART non-HAART also resulted in a lower TB incidence rate, the reduction did not reach statistical significance (p = 0.08). The findings in this study serve to validate similar findings in previous, more controlled studies, and demonstrate that it is possible for national programs to provide access to HAART under “real world” conditions, and in doing so, reduce the risk of the most common OI of adults living with HIV—TB [19]–[22].

In Brazil, it is customary for HIV treatment facilities to take over the care of newly diagnosed HIV-infected patients in the public sector, and thereafter, provide longitudinal comprehensive clinical management and ART, as indicated [25]. According to the WHO, 86% of diagnosed and eligible HIV-infected patients in Brazil were receiving ART in 2004, a proportion superior to that of low and middle income countries (e.g., Peru, 18%); and similar to that of developed countries in South America such as Argentina and Chile (both 100%) [26]. However, while access to ART remains very important, a significant impact in the reduction TB incidence for people living with HIV is unlikely unless a large proportion of the subset who are unaware of their HIV serostatus are tested, and national programs are able to deliver high quality comprehensive clinical care, including effective provision of ART to this population.

The patients who developed TB disease in our study had similar risk factors for TB as HIV-infected patients in other settings, and 66% eventually received HAART [1], [8], [11], [22], [34], [35]. Nevertheless, 11 (4%) patients developed TB disease despite receiving HAART. A higher proportion of patients with TB had median baseline CD4+ T-lymphocyte counts <200 cells/mm^3^ compared to patients without TB (57% vs. 37%, respectively. *Χ* ^2^, p = 0.01). This suggests that those who developed TB may have been more immunocompromised at baseline than patients remaining TB free; however, the median baseline absolute CD4+ T-lymphocyte count and baseline WHO clinical staging characteristics were not significantly different between the two groups. We cannot exclude the possibility, however, that the study lacked the power to detect subtle but clinically important immunological differences among groups. In areas of high TB prevalence, TB can occur at all levels of CD4+ T-lymphocyte count, though ART-naïve patients who develop TB frequently present with low CD4+ T-lymphocyte counts prior to TB diagnosis [36], [37]. Both HAART and non-HAART regimens have been shown to raise CD4+ T-lymphocyte counts and improve the clinical status of HIV-infected patients with and without concomitant OI [20], [38], [39]. This suggests that other factors may be affecting TB disease risk after initiating ART. Indeed, evidence exists to suggest that HAART is unable to fully restore the immune response to *Mycobacterium tuberculosis* in HIV-infected patients, leading to a chronically heightened TB risk over an increased lifespan [39]–[41]. Addressing this heightened risk poses a challenge for the future control of TB; however, growing evidence also suggests that this risk may be mitigated by early initiation of HAART with corresponding high levels of treatment adherence [24], [41], [42]. Fortunately, currently and widely recommended clinical guidelines for the treatment of HIV infection and TB disease are helping to eliminate an important negative factor from the TB/HIV equation—suboptimal ART. Because of the timeframe selected for this study, 1995–2001, it was not uncommon, early within this time interval, to document ART regimens consisting of single and dual ARV agents. Nevertheless, we saw a corresponding shift toward HAART (data not shown) in this study as scientific thought about ART gradually evolved after 1995 [41]. Attention to other factors such as facility infection-control, active case finding, and wider use of appropriately administered IPT could be instrumental in further reducing TB disease risk in HIV-infected individuals (Golub JE, et al. Tuberculosis incidence by HAART and isoniazid prophylactic therapy in HIV-infected patients in Rio de Janeiro, Brazil. XVI International AIDS Conference, 2006 Abstract no. MoPE0395) [5], [9], [23], [42]–[45]. On-going studies with regard to the use of IPT in HIV infection should elucidate the optimal use of this intervention (Samandari T, et al. Characteristics of people living with HIV-1 screened for isoniazid preventive therapy-Botswana, 2004–2005. AIDS 2006-XVI International AIDS Conference, 2006 Abstract no. MoAB010).

Finally, there is now reliable information showing that HIV-related morbidity and mortality in adults and children, including that due to TB disease co-morbidity, can be significantly reduced with the adjunctive use of CTM prophylaxis [46]–[48]. We found that the mortality rate and overall mortality of the cohort and of severely immunocompromised TB patients was comparable, if not lower, than that observed for HIV infected patients managed and reported in high income countries after 1995 [16], [17], [49]. And, while many factors clearly confound any mortality considerations in this study, the fact that 46% and 33% of the cohort and of TB patients, respectively, in the study received at least one dose of CTM probably contributed to favorable outcomes in these patients. As is probably the case in many other clinical settings globally, the use of CTM prophylaxis among HIV-infected populations with TB can clearly be improved and, in doing so, independently reduce patient morbidity and mortality.

Our study population specifically excludes children (age<18 years) and pregnant women. Due to the retrospective and observational design of this study, we were also unable to control for factors specific to (a) patients (e.g., adverse nutritional and psycho-social issues); (b) facilities (e.g., infection control measures); and, (c) programs [e.g., erratic pharmacy drug stocks and diagnostic rigor (e.g., low sensitivity and specificity of X-ray imaging)]. In addition, patients with only one clinic visit were explicitly excluded from the study, and we were unable to consistently determine the degree of patient adherence to therapeutic regimens from patient records. With regards to treatment adherence, however, WHO and others estimate adherence to be approximately 75% among patients on ART in Brazil [27], [50].

An additional limitation of our study was that the majority of the TB diagnoses recorded were made, primarily, on clinical grounds (i.e., presumed); and therefore, an alternative diagnosis to TB cannot be excluded. As the most common opportunistic disease of HIV-infected patients, however, the clinical experience of health care staff in facilities with large HIV patient populations probably lessens this possibility. In addition, TB misdiagnosis would only tend to underestimate the degree of TB incidence reduction by HAART.

Despite the limitations of the study and the fact that a proportion of study patients received sub-optimal ART before being switched to HAART, the significant reduction in TB disease observed is all the more remarkable. Our findings, however, are not applicable to pediatric patients, and should be interpreted in light of the study limitations.

We conclude that, in settings of unrestricted access to ART, HAART can have a significant impact on the TB incidence of HIV-infected patients developing. We found that, even under programmatic conditions, a statistically significant reduction in TB incidence of 80% was observed among HIV-infected patients receiving HAART in the public sector in Brazil compared to patients who remained ART-naïve. We demonstrate that, although broad access to ART remains an elusive goal of low and middle income countries, a national policy that drives the effective implementation of a program that manages HIV-infected patients using HAART is an initiative that is not only beneficial in reducing TB disease in HIV-infected patients, but also a feasible one.

## References

[1] Hopewell PC, Chaisson RE, Reichman LB, Hershfield ES (2000). Tuberculosis and human immunodeficiency virus Infection.. Tuberculosis, a comprehensive approach, 6^th^ ed.

[2] Grant AD, Djomand G, De Cock KM (1997). Natural history and spectrum of disease in adults with HIV/AIDS in Africa.. AIDS.

[3] Corbett EL, Churchyard GJ, Charalambos S, Samb B, Moloi V (2002). Morbidity and mortality in South Africa gold miners: impact of untreated HIV infection.. Clin Infect Dis.

[4] Nunn P, Brindle R, Carpenter L (1992). Cohort study on human immunodeficiency virus infection in patients with tuberculosis in Nairobi, Kenya.. Am J Resp Crit Care Med.

[5] Bucher HC, Griffith LE, Guyatt GH, Sudre P, Naef M (1999). Isoniazid prophylaxis for tuberculosis in HIV infection: a meta-analysis of randomized controlled trials.. AIDS.

[6] Daley CL, Small PM, Schecter GF, Schoolnik GK, McAdam RA (1992). An outbreak of tuberculosis with accelerated progression among persons infected with the human immunodeficiency virus.. N Engl J Med.

[7] Di Perri, Cruziani M, Danzi MC, Luzzati R, De Checchi (1989). Nosocomial epidemic of active tuberculosis among HIV-infected patients.. Lancet.

[8] Raviglione MC, Harries AD, Msiska R, Wilkinson D, Nunn P (1997). Tuberculosis and HIV: current status in Africa.. AIDS.

[9] Fitzgerald DW, Desvarieux M, Severe P, Joseph P, Johnson WD (2000). Effect of post-treatment isoniazid on prevention of recurrent tuberculosis in HIV-1-infected individuals: a randomised trial.. Lancet.

[10] Sonnenberg P, Murray J, Glynn JR, Shearer S, Kambashi B (2001). HIV-1 and recurrence, relapse, and reinfection of tuberculosis after cure: a cohort study in South African mineworkers.. Lancet.

[11] Bleed D, Dye C, Raviglione M (2000). Dynamics and control of the global tuberculosis epidemic.. Curr Opin Pulm Med.

[12] Godrey-Faussett P, Ayles H (2003). Can we control tuberculosis in high HIV prevalence settings?. Tuberculosis.

[13] Corbett EL, Watt C, Walker N, Maher D, Williams B (2003). The growing burden of tuberculosis: global trends and interactions with the HIV epidemic.. Arch Intern Med.

[14] World Health Organization (2002). Strategic framework to decrease the burden of TB/HIV..

[15] World Health Organization (2002). Interim policy on collaborative TB/HIV activities..

[16] Palella FJ, Delaney KM, Moorman AC (1998). Declining morbidity and mortality among patients with advanced HIV infection.. N Engl J Med.

[17] Hung CC, Chen MY, Hsiao CF, Hsieh SM, Sheng WH (2003). Improved outcomes of HIV-1-infected adults with tuberculosis in the era of highly active antiretroviral therapy.. AIDS.

[18] Girardi E, Palmieri F, Cingolani A, Ammassari a, Petrosillo N (2001). Changing clinical presentation and survival in HIV-associated tuberculosis after highly active antiretroviral therapy.. J Acquir Immune Defic Sydr.

[19] Dheda K, Lampe FC, Johnson MA, Lipman MC (2004). Outcome of HIV-associated tuberculosis in the era of highly active antiretroviral therapy.. J Infect Dis.

[20] Badri M, Wilson D, Wood R (2002). Effect of highly active antiretroviral therapy on incidence of tuberculosis in South Africa: a cohort study.. Lancet.

[21] Girardi E, Antonucci G, Vanacore P, Libanore M, Errante I (2000). Impact of combination antiretroviral therapy on the risk of tuberculosis among persons with HIV infection.. AIDS.

[22] Jones JL, Hanson MS, De Cock KM (2000). HIV-associated tuberculosis in the era of highly active antiretroviral therapy.. Int J Tuberc Dis.

[23] Santoro-Lopes G, Felix de Pinho AM, Harrison LH, Schecter M (2002). Reduced risk of tuberculosis among Brazilian patients with advanced human immunodeficiency virus infection treated with highly active antiretroviral therapy.. Clin Infect Dis.

[24] Williams BG, Dye C (2003). Antiretroviral drugs for tuberculosis control in the era of HIV/AIDS.. Science.

[25] Levi GC, Vitoria MA (2002). Fighting against AIDS: the Brazilian experience.. AIDS.

[26] World Health Organization (2007). Global tuberculosis control: surveillance, planning, financing. WHO report 2007..

[27] World Health Organization (2004). “3 by 5” progress report..

[28] Fleiss JL, Levin B, Myunghee CP, Shewart WA, Samuel SW (2004). Determining sample sizes needed to detect a difference between two proportions.. 2^nd^ ed.

[29] World Health Organization (1990). Acquired immunodeficiency syndrome: interim proposal for a WHO staging system for HIV infection and disease.. Wkly Epidemiol Rec.

[30] Ministério da Saúde do Brasil, Programa Nacional de DST e Aids, Secretaria de Vigilância em Saúde (2006). Recomendacões para terapia anti-retroviral em adultos e adolescentes infectados pelo HIV (versão preliminar)..

[31] Ministério da Saúde do Brasil, Secretaria de Políiticas de Saúde, Departamento de Atencion Básica (2002). Manual técnico para o controle da tuberculose: cadernos de atenção básica, 6^th^ ed..

[32] Lipman M, Breen R (2006). Immune reconstitution inflammatory syndrome in HIV.. Curr Opin Infect Dis.

[33] Shelburne SA, Visnegarwala F, Darcourt J, Graviss EA, Giordano TP (2005). Incidence and risk factors for immune reconstitution inflammatory syndrome during highly active antiretroviral therapy.. AIDS.

[34] Selwyn PA, Hartel D, Lewis VA, Schoenbaum EE, Vermund SH (1989). A prospective study of the risk of tuberculosis among intravenous drug users with human immunodeficiency virus infection.. N Engl J Med.

[35] Badri M, Pulerwitz T, Wood R, Maarten G (2002). Tuberculosis should not be considered an AIDS-defining illness in areas with high tuberculosis prevalence.. Int J Tuberc Lung Dis.

[36] Mukadi Y, Perriëns JH, St. Louis ME, Brown C, Prignot J (1993). Spectrum of immunodeficiency in HIV-1-infected patients with pulmonary tuberculosis in Zaire.. Lancet.

[37] Wolday D, Hailu B, Girma M, Hailu E, Sanders E (2003). Low CD4+ T-lymphocyte T-cell and high viral load precede the development of tuberculosis disease in a cohort of HIV-positive Ethiopians.. Int J Tuberc Lung Dis.

[38] Eron JJ, Benoit SL, Jemsek J (1995). Treatment with lamivudine, zidovudine or both in HIV- positive patients with 200–500 CD4+ T-lymphocyte cells per cubic millimeter.. N Engl J Med.

[39] Lawn SD, Bekker LG, Wood R (2005). How effectively does HAART restore immune response to mycobacterium tuberculosis? Implications for tuberculosis control.. AIDS.

[40] Lienhardt C, Rodrigues LC (1997). Estimation of the impact of human immunodeficiency virus infection on tuberculosis: tuberculosis risk revisited.. Int J Tuberc Dis.

[41] Jorda R, Gold L, Cummins C, Hyde C (2002). Systematic review and meta-analysis of evidence for increasing numbers of drugs in antiretroviral combination therapy.. Br Med J.

[42] Maher D, Borgdorff M, Boerma T (2005). HIV-related tuberculosis: how well are we doing with current control efforts?. Int J Tuberc Lung Dis.

[43] World Health Organization (1999). Preventive therapy against tuberculosis in people living with HIV.. Wkly Epidemiol Rec.

[44] World Health Organization 2004 Isoniazid preventive therapy. Report of a “lessons learnt” workshop of the six PROTEST pilot projects in Malawi, South Africa, and Zambia Geneva World Health Organization (WHO/HTM/TB/2004.336)In:27 30

[45] World Health Organization (2006). Tuberculosis infection control in the era of expanding HIV care and treatment: addendum to WHO guidelines for the prevention of tuberculosis in health care facilities in resource-limited settings..

[46] Wiktor SZ, Sassan-Morokro M, Grant AD, Abouya L, Karon JM (1999). Efficacy of trimethoprim-sulphamethoxazole prophylaxis to decrease morbidity and mortality in HIV-1-infected patients with tuberculosis in Abidjan, Cote d'Ivoire: a randomised controlled trial.. Lancet.

[47] World Health Organization (2002). Scaling up antiretroviral therapy in resource-limited settings: Guidelines for a public health approach..

[48] Chintu C, Bhat GJ, Walker AS, Mulenga V, Sinyinza F (2004). Co-trimoxazole as prophylaxis against opportunistic infections in HIV-infected Zambian children: a double-blind randomised placebo-controlled trial.. Lancet.

[49] Mocroft A, Vella S, Benfield TL, Chiesi A, Miller V (1998). Changing patterns of mortality across Europe in patients infected with HIV-1.. Lancet.

[50] Carmody ER, Ellie R, Diaz T, Starling P, Santos AP (2003). An evaluation of antiretroviral HIV/AIDS treatment in a Rio de Janeiro public clinic.. Trop Med Int Health.

